# Supplementary material for: Three-dimensional bulk electronic structure of the Kondo lattice CeIn3 revealed by photoemission
Source: Sci Rep. 2016 Sep 19;6:33613. doi: 10.1038/srep33613 (PMC5027528; doi:10.1038/srep33613)
Supplement: Supplementary Information [file srep33613-s1.docx]

**Three-dimensional bulk electronic structure of the Kondo lattice CeIn_3_ revealed by photoemission**

Yun Zhang^1,2*^, Haiyan Lu^1^, Xiegang Zhu^1^, Shiyong Tan^1^, Qin Liu^1^, Qiuyun Chen^1^, Wei Feng^1^,

Donghua Xie^1^, Lizhu Luo^1^, Yu Liu^4,5^, Haifeng Song^4,5^, Zhengjun Zhang^3^, Xinchun Lai^1*^

*^1^Science and Technology on Surface Physics and Chemistry Laboratory, Mianyang 621907, China*

*^2^Department of Engineering Physics,* *Tsinghua University, Beijing 100084, China*

*^3^School of Materials Science and Engineering, Advanced Materials Laboratory, Tsinghua University, Beijing 100084, China*

*^4^Laboratory of Computational Physics, Institute of Applied Physics and Computational Mathematics, Beijing 100088, China*

*^5^CAEP Software Center for High Performance Numerical Simulation, Beijing 100088, China*

**Supplementary Information**


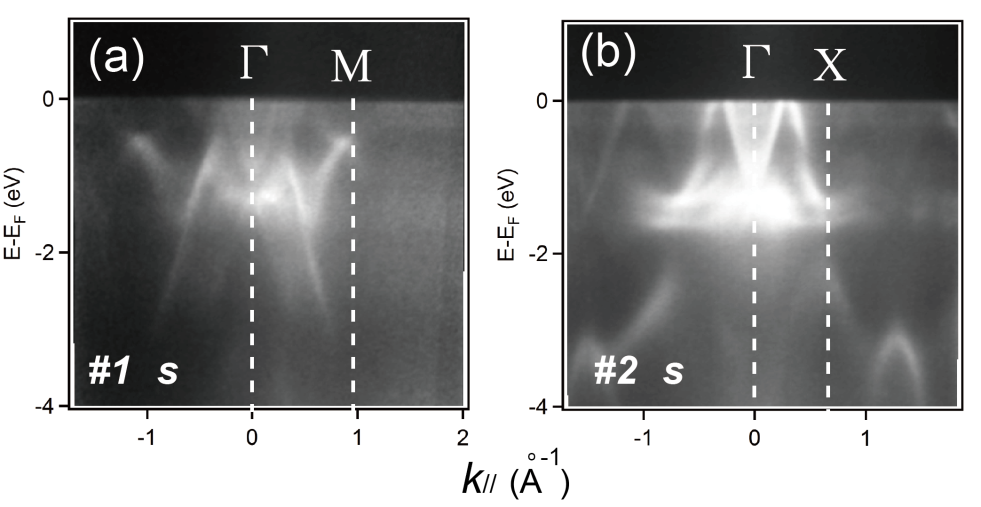


Figure S1. Valence band structure of CeIn_3_. (a) and (b) Photoemission intensity plots taken using 542 eV *s*-polarised light along the Γ-M and Γ-X directions, respectively.
 Figure S1 shows the ARPES spectra along the Γ-M and Γ-X directions. Two nearly non-dispersive *f* bands are observed around E_F_. If we change the relative intersection angle between the surface of the sample and the light source, the two flat *f* bands seem constant over all angles and over different photon energies. Namely, the two flat bands are apparently momentum and photon-energy independent. In fact, if hybridization between *f* electrons and conduction electrons occurs, then the *f* bands should not be visible in some of the momentum space around E_F_. The region where hybridization occurs should have an energy gap, and the *f* band should only be partially observed, as is shown in Fig. 4(c) in the main text. We propose that the *f* electrons in CeIn_3_ primarily remain nearly localized at ambient pressure and low temperature.
